# A Handbook of Surgical Pathology

**Published:** 1890-03

**Authors:** 


*A Handbook of Surgical Pathology.* Second Edition. By W. J. WALSHAM, M.B., F.R.C.S.; assisted by D'ARCY POWER, M.A., M.B., F.R.C.S. Pp. 634. London: BAILLIÈRE & CO. 1890.

We have only good to say of this work. It is a practical embodiment of the best of all possible plans of teaching—the objective. It is an exposition of surgical pathological principles as illustrated by pathological specimens available to students in the St. Bartholomew's Hospital Museum. There is only one cause for regret; and that is, that the museum is not complete. The remark appended to so many descriptions, "No specimen in the museum," makes us long for such a work based on the Hunterian collection, or one which by mutual exchanges and selections could be made even more perfect than that.

